# Distress as a mediator for pain and activities of daily living in older adults with fibromyalgia

**DOI:** 10.3389/fmed.2022.1033936

**Published:** 2022-12-14

**Authors:** Caitlin Gower, Jennifer Trevitt, Barbara J. Cherry, Laura Zettel-Watson

**Affiliations:** ^1^Fibromyalgia and Chronic Pain Center, California State University, Fullerton, Fullerton, CA, United States; ^2^Department of Psychology, California State University, Fullerton, Fullerton, CA, United States; ^3^Aging Studies Academic Program, California State University, Fullerton, Fullerton, CA, United States

**Keywords:** depression, fibromyalgia, anxiety, chronic pain, mediation, distress, activities of distress

## Abstract

**Introduction:**

Pain, distress, and activities of daily living impact the lives of those with chronic pain. This study investigated distress (depressive symptoms, anxiety) on the relationship between pain (intensity and pain interference) and activities of daily living in individuals with fibromyalgia while controlling for age.

**Methods:**

The current cross-sectional investigation focused on data from 123 men and women with fibromyalgia. Pain intensity, pain interference and anxiety were measured on 0-10 Likert type scales from the National Fibromyalgia Assessment Questionnaire. Depressive symptoms were assessed using the Beck Depression Inventory II. Activities of daily living (basic, instrumental) were measured with the Physical Activity Inventory Scale.

**Results:**

It was hypothesized that the relationships between pain intensity and pain interference and activities of daily living in individuals with fibromyalgia would be mediated by the construct of distress while controlling for age. Mediation significantly occurred in both models as predicted. However, those who were older reported lower levels of pain intensity and distress than their younger counterparts, which may be related to time since diagnosis or other factors.

**Discussion:**

Results of this study suggest that individuals with chronic pain conditions would benefit from treatment options which address distress, specifically depressive symptoms and anxiety.

## Introduction

Fibromyalgia (FM) is a chronic widespread pain condition, affecting approximately 2.7% of the worldwide population and 1.75% of the American population, with the prevalence rising with age (1–5). While FM studies tend to recruit older women as participants, FM can affect anyone around the world at any age range, of any gender (6). FM is characterized by chronic pain and fatigue, which impact the cognitive abilities, physical abilities, and mood state of those affected (7).

In addition, typical comorbidities include chronic fatigue syndrome, irritable bowel syndrome, and post-traumatic stress disorder, to name a few (2, 8). Often called the invisible disease, there is no outward manifestation of the syndrome (9). This lack of visible evidence of a chronic and debilitating illness can make doctors, friends, family, and even patients themselves doubt their own diagnosis and become discouraged during the diagnostic process (10). One way to improve this is education and understanding of the syndrome, and to be able to relate it to other conditions like chronic fatigue syndrome or long COVID. Letting folks know they are not alone, and that we are still working on understanding FM and its treatments is essential.

### Symptom clusters

Symptom clusters are defined as groupings of symptoms often felt by people with FM, relatively independent of other symptoms (11–13). These include anxiety, depression, fatigue, pain, and stiffness in the morning, among others. Symptom clusters are interesting because they show variability within the FM population. Some commonly named symptom clusters in FM include labels such as somatic, distress, fibromyalgia core, dyscognition, and sleep problems (13). Of particular interest is the symptom cluster that encompasses anxiety and depression, labeled distress.

Distress is important because it combines two of the most common comorbid emotional conditions with FM: anxiety and depression. Depression is well studied in individuals with FM, with some suggesting that FM and depression are both representations of affective spectrum disorder (14). The prevalence of patients with FM also experiencing a depressive episode is 20–86%, with depression increasing the risk of developing FM and vice versa (15, 16). Depression can lead to various emotional and physical problems and can decrease one’s ability to function at work or at home (17). Older adults who suffer from depression can experience severe consequences including an increased risk of suicide, increased burden of physical illness, and higher functional impairment (18).

If the depressive symptoms of FM are treated, the physical symptoms of pain and inflammation will often subside (7). Andrade et al. also pointed out that the reverse is true. Additionally, major depressive disorder seems to be equally predicted by either familial major depressive disorder or by a family history of FM (14). Moreover, depression can also be a barrier to seeking treatment in individuals with chronic pain (19).

The other aspect of distress, anxiety, affects up to one-third of patients with FM (20). It has been found that anxiety affects perception of pain and somatization of symptoms in people with FM (21). FM patients with higher levels of anxiety also report higher pain intensity and pain interference than those with lower levels of anxiety (22). Further, levels of anxiety and depression are higher in individuals with FM when compared to healthy controls, and this distress increases with age (23). In short, anxiety and depression, representing the construct of distress, are important to include when studying FM.

FM is a chronic pain syndrome of unknown etiology, and treating one symptom may relieve others. To understand FM more thoroughly, the following sections explore the relationship between pain, distress (depression and anxiety), and activities of daily living (ADLs).

### Pain and activities of daily living in fibromyalgia

Pain has been shown to influence ADLs in individuals with FM, which can best be seen in the ability to accomplish goals and participate in activities for those affected. Pain from FM is pervasive, affecting people’s sexuality, cognitive functioning, and ability to take care of very basic needs through ADLs (24–26).

The relationship between pain and ADLs is negative, strong, and bidirectional in those with FM, such that as pain increases, the ability to perform ADLs decreases (27). In Dailey et al. (28) study on pain and perceived physical functioning in individuals with FM, researchers found that pain significantly predicted perceived physical functioning. Those with higher pain scores had lower perceived physical functioning (28).

### Pain and distress in fibromyalgia

Woo (29) addressed depression and anxiety and their relationships with pain intensity. He stated that anxiety produces fear and worry and discusses catastrophizing, hypervigilance, and fear avoidance, and how they all stem from anxiety and pain (29). Pain is also related to higher levels of emotional distress and fatigue in individuals with FM (30). Depression and anxiety add to pain because they may increase the likelihood of social isolation and kinesophobia, or the fear of movement due to pain (31, 32). Additionally, the pathophysiology of depression and anxiety may use the same neurotransmitters that affect pain in the brain (33). Therefore, this relationship should be investigated further, especially in individuals with FM.

#### Pain intensity and distress in fibromyalgia

Certain antidepressants like SSRIs and SNRIs have been shown to treat both the painful symptoms of FM as well as depression (34, 35). This suggests that pain intensity and depression in individuals with FM have a unique relationship. There is a bidirectional temporal relationship between FM symptoms like pain and depression (16). This means that those with FM are at higher risk of developing depression over time and those with depression are at higher risk of developing FM over time. Additionally, those with higher pain scores tend to have higher levels of depression (28).

Pain intensity is also related to anxiety in individuals with and without FM. Like depression, anxiety shares the pathway of pain in the brain. In one study, it was found that FM patients and their pain have a unique relationship with pain intensity and anxiety, with FM patients who had higher pain intensity also having higher anxiety levels (36). In this study, the other groups that were tested did not share this relationship. There is also evidence of high levels of anxiety related to FM patients’ heightened perception of pain (21).

#### Pain interference and distress in fibromyalgia

Pain interference may be defined as the extent to which pain interferes in the engagement of physical, cognitive, emotional, and recreational activities, as well as sleep and enjoyment in life (37). Pain interference is routinely measured in individuals who are recovering from surgery, a severe injury, or suffering from a chronic pain condition (38). Specifically, in the FM population, high levels of pain interference have been tied to lower levels of anxiety, depression, and lower scores on ADLs scales (39).

### Activities of daily living and distress in fibromyalgia

Distress and its relationship with ADLs in individuals with chronic pain is important to examine because individuals with chronic pain are not a homogeneous group. Those with chronic pain differ in their ability to perform ADLs among themselves and when compared to the general population (40). Depression and anxiety negatively affect chronic pain self-management and only 37% of those with depression and anxiety are receiving treatment for these conditions (19, 41). Additionally, depression can influence ADLs and functional abilities in older adults (42, 43). However, little research has been done on anxiety and depression, and how together they might influence the relationship between pain and ADLs in individuals with FM experiencing distress.

#### Activities of daily living and depression in fibromyalgia

For older adults, depression may be one of the strongest risk factors for needing assistance in one or more basic/instrumental ADLs in later life, and at least in Norway, depression is being targeted for preventative purposes in older adults (44). In many studies, higher levels of depression were associated with lower levels of perceived physical functioning or ADLs. This can be seen in Cipher and Clifford (45) paper on depression, perceived physical functioning, and quality of life in long-term care. Here, the researchers concluded that depression, pain, and ADLs were all interrelated and highly important for quality of life for those in long-term care.

#### Activities of daily living and anxiety in fibromyalgia

ADLs and anxiety also have an interesting relationship. While the majority of the research on ADLs and pain in the FM population has focused on depression, anxiety also plays a vital role. For example, Dailey et al. (28) discuss factors influencing perceived physical functioning in women with FM. They discovered that fear, depression, anxiety, and catastrophizing all factor into this relationship. In addition, Costa et al. (46) state that impaired gait and balance, which are important to ADLs, are associated with high levels of anxiety, pain, and depression.

Additionally, anxiety in individuals with FM is associated with other things that influence ADLs indirectly, like pain catastrophizing, hypervigilance, and fear avoidance (29). Pain catastrophizing is defined as dwelling on the worst possible outcomes (29). Hypervigilance is defined in this setting as being extremely aware of pain and being unable to distract oneself from pain-related stimuli (29). Lastly, fear avoidance is defined here as avoidance of movement or activities based on fear of pain (kinesophobia) (29). While anxiety has not been studied extensively in the past in relation to ADLs and pain in the FM population, constructs stemming from anxiety (e.g., kinesophobia, hypervigilance, and pain catastrophizing) have. Higher levels of these constructs have been shown to be related to higher levels of disability and a decreased ability to be functionally independent, which is what ADLs measure.

### Previous study on pain, activities of daily living, and depression in fibromyalgia

One study went into detail on the relationship between pain, ADLs, and depression. This research, conducted by Steiner et al. (47), followed 216 participants over a 36-week period. They measured depression, pain intensity, and perceived physical function at baseline, 12, 24, and 36 weeks. Using structural equation modeling, they used longitudinal mediation to determine whether depression mediated the relationship between ADLs and pain in individuals with FM over time [the full list of inclusion/exclusion criteria for this study can be found in Ang et al. (48)]. They found that depression was a partial mediator of the relationship between physical functioning (ADLs) and pain intensity for their sample at all four time points, with higher levels of depression being related to lower levels of ADLs and higher levels of pain. Depression was a partial mediator in this relationship; that is, pain had some residual direct effects on ADLs even after depression was introduced into the model, instead of depression explaining the relationship entirely (49). Steiner et al. (47) suggest that treating depression could be a viable option in the pursuit of improving pain symptoms and ADL abilities for those with FM.

While this is a very important and promising finding for those with FM who have difficulties in these categories, these findings can be expanded upon. Steiner et al. (47) did not include anxiety in their measures, even though anxiety has been shown to play an important part in the relationship between pain and ADLs. Steiner et al. (47) also used the Personal Health Questionnaire Depression Scale (PHD-8) and the pain intensity portion of the Brief Pain Inventory for pain. It is important to retest this relationship while using measures specifically validated for use in older adults with FM to see whether the relationship still exists. Thus, the current study includes the Beck Depression Inventory II (BDI-II) for depression and the PAI for ADLs.

Although Steiner et al. (47) used longitudinal data, they did not report many longitudinal findings. Instead, they analyzed the mediation models for each time point cross-sectionally, while adjusting for each variable’s measure at the previous time point. The mediation models for each time point indicated that depression was a significant partial mediator in the relationship between pain and ADLs. The results also showed that the means for each variable and the strength of the relationships changed across assessment points, but the mediating relationship of depression remained the same. Depression at any time point was a significant partial mediator of the pain intensity and physical functioning relationship at that same time point.

Since things like ADL difficulty, distress (anxiety and depression), and pain persist despite treatment in individuals with FM who are older, it is important to investigate the relationships between these variables. Because of these factors, people with FM have more difficulty with ADLs than healthy individuals.

### Current study

The literature shows associations between pain, ADLs, and distress; higher levels of pain are associated with lower levels of functionality, measured through ADLs. It has also found that higher levels of pain are related to higher levels of depression and anxiety. Moreover, higher levels of anxiety and depression are related to lower levels of functionality, measured through ADLs. Steiner et al. (47) found that depression partially mediates the relationship between pain and ADLs; however, distress, which encompasses both anxiety and depression, should be explored, because anxiety is important when studying secondary pain effects. Pain interference should also be investigated, above and beyond pain intensity.

With a better understanding of the links between the symptoms of this syndrome and other factors, chronic pain support groups, physicians, and researchers can offer specialized help for patients. While treating pain and other symptoms of FM may be difficult, treating depression and anxiety is possible and reversible in older adults and in the chronic pain population (42, 50). If there is a mediating relationship between pain, distress, and ADLs, treating the mood disorders may help with the physical limitations of FM.

The current study thus focused on the relationship between pain, distress, and ADLs. The independent variables in this study revolve around pain, specifically pain intensity and pain interference; the dependent variable in this study is ADLs and the mediator is distress. Pain intensity is measured by a 10-point Likert-type scale (NFAQ). Pain interference is measured by a 10-point Likert-type scale (NFAQ). ADLs are measured by the Physical Activity Inventory (PAI) total score. Depressive symptoms were measured with the Beck Depression Inventory (II) (BDI-II) total score, and anxiety was measured by a 10-point Likert-type scale (NFAQ). Distress was calculated from depressive symptom scores and anxiety scores.

The overarching hypothesis for the first model was: the mediating variable, distress, would help explain the relationship between pain intensity and ADLs in individuals with FM, even after controlling for potential extraneous variables like age, gender, and income. The overarching hypothesis for the second model was: the mediating variable, distress, would help explain the relationship between pain interference and ADLs in individuals with FM, even after controlling for age.

## Materials and methods

### Participants

The data were collected by the Fibromyalgia and Chronic Pain Center, at California State University, Fullerton, at four time points: 2008, 2010, 2012, and 2014. Data were collected from 123 men and women with FM aged 50–85 (*M* = 59.66, *SD* = 7.35). Participants were excluded from parts of the study if it was physically unsafe for them to participate or they scored lower than 25 out of 30 on the Mini-Mental screening for dementia, as the parent study included physical and cognitive performance measures. For the purposes of this study, only first-time scores were used. That is, not every participant participated at every time point, with some participating in 2008, 2010, 2012, or 2014 for the first time. This study only used the scores of the participants from their first wave, which means that the year in which their data were collected may vary.

### Measures

Pain, anxiety, depression, ADLs and demographics were all measured in this study. Distress was calculated from anxiety and depression.

#### Pain intensity

Pain intensity was measured on a 0–10 Likert-type scale from the National Fibromyalgia Association Questionnaire (NFAQ) (51). The pain question read, “Please circle the number that best describes your experience with [pain] ON AVERAGE during the past week,” with higher numbers indicating higher pain intensity. The NFAQ itself has been shown to have high content validity, stability, and high internal consistency with a Cronbach’s alpha of α = 0.88 (51, 52).

#### Pain interference

Pain interference was measured on a 0–10 Likert-type scale from the National Fibromyalgia Association Questionnaire [NFAQ; (51)]. The pain interference question read, “How much “bodily pain” have you generally had during the past 4 weeks (while doing normal ADLs)?,” with higher numbers indicating more pain interference. The NFAQ itself has been shown to have high content validity, stability, and high internal consistency with a Cronbach’s alpha of α = 0.88 (51, 52).

#### Anxiety

Anxiety was measured on a 0–10 Likert scale pulled from the NFAQ (51). The anxiety question read, “Please circle the number that best describes your experience with [anxiety] ON AVERAGE during the past week,” with higher numbers indicating more anxiety. As stated above, the NFAQ itself has been shown to have high content validity, stability, and high internal consistency with a Cronbach’s alpha of α = 0.88 (51, 52).

#### Depression

To measure the participants’ depression levels, the BDI-II was used. The Beck Depression Inventory-II (53) has 21 total items, and has been validated with both older adults and adults with FM. Each answer has a 0–3 rating scale, with a total possible score of 63. The interpretation of depression score is as follows: (0–13) is minimal; (14–19) is mild; (20–28) is moderate; (29–63) is severe. This scale has high internal consistency of α = 91 and high 1-week test-retest reliability *r* = 0.93 (54).

#### Distress

Distress was calculated from the anxiety scale and the BDI-II. As stated above, anxiety is on a Likert-type scale with a maximum score of 10 and the BDI-II is on a 63-point scale. To create the variable of distress, the BDI-II total score for each participant was divided by 6.3 in order for the two scales to be weighted equally. Then, the sum of the anxiety score and the modified depression score were divided by 2 for each participant, resulting in a distress score from 0 to 10.

#### Activities of daily living

ADLs are measured through the 12-item Physical Ability Impact Scale (PAI) which was adapted from the longer Composite Physical Functioning Scale (CPF) (55, 56). This 12-item scale assessed functional limitations and ADLs. Participants were instructed to indicate their perceived ability to do certain tasks during the span of a week. Examples of some of these tasks included “Take care of own personal needs (e.g., dressing yourself),” “Walk 1 mile (12–14 blocks),” and “Do light household activities (e.g., cooking, dusting, washing dishes, sweeping a walkway).” Response scales for each item ranged from 0 to 4, with 0 being “cannot do at all,” 1 being “cannot do without help,” 2 being “can do with a lot of difficulty,” 3 being “can do with some difficulty,” and 4 being “can do without difficulty.” Higher scores on the PAI indicated less functional limitation, with a composite score of 44–48 indicating high functioning. The PAI has been shown to have adequate concurrent validity with the Fibromyalgia Impact Questionnaire, high internal consistency with a Cronbach’s alpha of α = 0.93 and a high test-retest reliability (*r* = 0.98) (52).

#### Demographics

Baseline age, education, gender, income, and employment status were all gathered in a demographic worksheet where participants were asked basic information. These demographic questions were part of the NFAQ (51).

### Procedures

Prior to any data collection, the California State University, Fullerton Institutional Review Board (CSUF IRB) granted approval for research with human subjects. This was done before each wave of data collection, and everyone collecting data participated in the Collaborative Institutional Training Initiative (CITI) Program. Informed consent was reviewed on assessment day with research assistants. Investigators then answered any participant questions.

Participants were recruited through email, phone calls, flyers, and word of mouth. Once a person indicated interest in the study, the Fibromyalgia and Chronic Pain Center team assessed their eligibility *via* phone [complete inclusion/exclusion criteria can be found in Cherry (57)]. Eligible participants were scheduled to come in for one 2–3-h session California State University, Fullerton. Prior to the in-person testing date, participants were mailed packets with informed consent, and various questionnaires. On assessment day, participants were asked to fill out packets which contained the BDI-II, the PAI, and the questionnaire containing the pain scale, demographics, and anxiety scale.

Participants were not financially compensated for their time; instead, they were invited back for a feedback conference, during which “report cards” were handed out with details about their individual physical/cognitive performance (i.e., the primary outcomes of the parent study). Resources and workshops were made available to all participants.

## Results

### Mediation

To assess the relationships between pain intensity, pain interference, distress, and ADLs in individuals with fibromyalgia while controlling for age, two mediation analyses were conducted.

### Assumptions

Assumptions that must be met for mediation analyses are the same as the general linear model: linearity, normality, homogeneity of error variance, and independence of errors. These assumptions were checked in SPSS and no adjustments were needed prior to testing the mediation model. Missing data were handled with the linear trend at point method available in SPSS prior to performing the mediation. This means that the missing values were replaced with the linear trend for that point, with the existing series being regressed on a variable scaled 1 to *n*. Missing variables were determined to be missing completely at random (MCAR) prior to replacement.

### Software

Mediation analyses were conducted in SPSS version 26 following the method outlined by Hayes (58).

### Procedure

Preliminary analyses were conducted to determine which covariates, in addition to age, would be appropriate for use in the models. *T*-tests for gender differences in pain intensity, pain interference, distress, and ADLs yielded no significant mean differences in scores between men and women. This indicates that the analyses could be run with males and females in the same models. Pain intensity and pain interference were significantly correlated (*r* = 0.664, *p* < 0.01), indicating that it would be beneficial to run the analyses of pain intensity and pain interference as two separate mediation models. We refer to the pain intensity model as Model 1, and the pain interference model as Model 2.

Demographic characteristics can be found in Tables 1, 2 shows the observed scores for pain intensity, pain interference, distress, and ADLs. As shown in Tables 3, 4, older participants reported less pain intensity and less distress than their younger counterparts. Additionally, as pain intensity/interference scores increased for participants, so did distress scores. Lastly, as pain and distress scores increased for participants, ADL scores decreased, indicating less functionality for those experiencing more pain intensity/interference and distress.

**TABLE 1:** Demographics.

Characteristics	*N*	%	*M*	*SD*
**Gender**				
Male	8	6.5		
Female	115	93.5		
Age (years)			59.66	7.25
**Time since diagnosis (years)**				
<1 yr	4	3.3		
1–5 yrs	26	21.3		
6–10 yrs	38	31.1		
11–15 yrs	28	23.0		
16–20 yrs	13	10.7		
>20 yrs	13	10.7		
**Level of education**				
Some high school	2	1.6		
High school or GED	7	5.7		
Trade/technical/CC/some college	45	36.6		
College degree	32	26		
Professional/graduate degree	37	30.1		
**Ethnicity**				
Hispanic	14	11.4		
White	106	89.1		
Black	2	1.7		
Asian/Pacific Islander	1	0.8		
American Indian	1	0.8		
Multiracial	6	5.0		
Other	4	3.3		
**Income (Dollars a year)**				
<9,000–39,999	36	31.3		
40,000–69,999	29	25.3		
70,000–99,999	27	23.5		
100,000– > 200,000	23	20		
**Employment status**				
Retired	31	25.4		
Working full time	23	18.9		
Working part time	17	13.9		
On temporary leave	1	8.0		
Looking for work	5	4.1		
Permanently disabled	27	22.1		
Keeping house	12	9.8		
Other	6	4.9		

**TABLE 2:** Descriptives observed for pain intensity, pain interference, distress, ADLs and age, *N* = 123.

Variable	Min	Max	Mean	SD
Pain intensity	0	10	6.195	2.249
Pain interference	2	5	3.593	0.808
BDI-II	0	45	17.950	9.485
Anxiety	0	10	4.768	2.971
Distress	0	8.57	3.809	2.004
ADLs	6	48	34.153	9.260

Pain intensity, pain interference, anxiety, and distress had a possible range of (0–10). BDI-II had a possible range of (0–63), and ADLs had a possible range of (0–48).

**TABLE 3:** Correlation matrix for pain intensity, distress, ADLs and age, *N* = 123.

Variable	1	2	3	4
1. Pain intensity	1	−0.452**	0.229*	−0.213*
2. ADLs		1	−0.348**	–0.051
3. Distress			1	−0.203*
4. Age				1

*p < 0.05, **p < 0.01 (two-tailed).

**TABLE 4:** Correlation matrix for pain interference, distress, ADLs and age, *N* = 123.

Variable	1	2	3	4
1. Pain interference	1	−0.519**	0.294**	–0.101
2. ADLS		1	−0.348**	–0.051
3. Distress			1	−0.203*
4. Age				1

*p < 0.05, **p < 0.01 (two-tailed).

It was proposed that pain intensity (*x*) would be negatively associated with ADLs (*y*). It was also proposed that pain intensity (*x*) would be positively associated with distress (*m*), which would be negatively associated with ADLs (*y*) while controlling for age (*c*_1_). This hypothesis is mirrored in the second model, with pain interference (*x*) being negatively associated with ADLs (*y*), and distress (*m*) mediating that relationship while controlling for age (*c*_1_).

### Model 1: Pain intensity mediation

**FIGURE 1:**
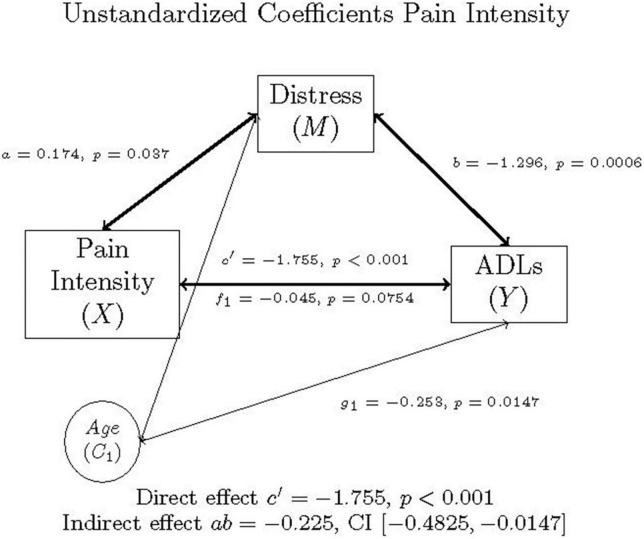
Mediation model of pain intensity, distress, ADLs, and age in individuals with FM, as well as the direct effect, indirect effect, and confidence interval using unstandardized (b) coefficients.

In the first model, the proposed mediator, distress (*m*), was regressed on pain intensity (*x*) to produce *a.* ADLs (*y*), was regressed on both distress (*m*) and pain intensity (*x*), which yields both *b* and *c’*, respectively, while controlling for age (*c*_1_). The relationship between pain intensity (*x*) and ADLs (*y*), controlling for age (*c*_1_), is represented by path *c*, found in Figure 1. The paths *f*_1_ and *g*_1_ represent the relationships between the covariate age (*c*_1_) and distress (*m*) and ADLs (*y*), respectively.

The PROCESS output is summarized in Tables 5, 6, and graphically represented in Figure 1. As illustrated in these tables and figures, mediation occurred in this model.

**TABLE 5:** Model coefficients for the pain intensity, distress, ADLs simple mediation model with covariate of age.

Antecedent		Consequent								
		*M* (Distress)					*Y* (ADLs)			
		β	*b*	*SE*	*p*		β	*b*	*SE*	*p*
X (pain intensity)	*a*	0.195	0.174	0.080	0.037	*C’*	–0.426	–1.755	0.331	<0.001
M (distress)		−	−	–	−	*b*	–0.281	–1.296	0.370	<0.001
C_1_ (age)	*f*_1_	–0.161	–0.045	0.025	0.075	*g*_1_	–0.196	–0.253	0.102	0.0147
Constant	*i*_*M*_	−	5.397	1.673	0.0016	*i*_*Y*_	−	65.076	7.071	<0.001
		*R*^2^ = 0.0774					*R*^2^ = 0.2958			
		*F*(2, 120) = 5.0303, *p* = 0.0080					*F*(3, 119) = 16.6596, *p* < 0.001			

**TABLE 6:** Mediation effects of distress on the relationship between pain intensity and ADLs controlling for age, *N* = 123.

			95% CI	
Effect	β	*b*	Lower	Upper
Total (*c* path)	–0.481	–1.980	–2.652	–1.309
Direct (*c*’ path)	–0.426	–1.755	–2.409	–1.100
Indirect (mediation, *ab* path)	–0.055	–0.225	–0.483	–0.015

Multiplying *a* and *b* yields the indirect effect, *ab* = 0.174 (-1.296) = –0.225. A 95% bootstrap confidence interval did not cross zero. The direct effect was *c’* = –1.755. The total effect was determined by adding the direct and indirect effects together: *c* = *c’* + *ab* = –1.755 + –0.225 = –1.980. Since the confidence interval did not cross zero, and everything is significant, the model is considered a good fit for the data. The Sobel Test indicated that mediation did not occur (Test statistic = 1.847, *p* = 0.0647), though Hayes (58) states that the Sobel Test may be too conservative to detect real mediation effects.

### Model 2: Pain interference mediation

**FIGURE 2:**
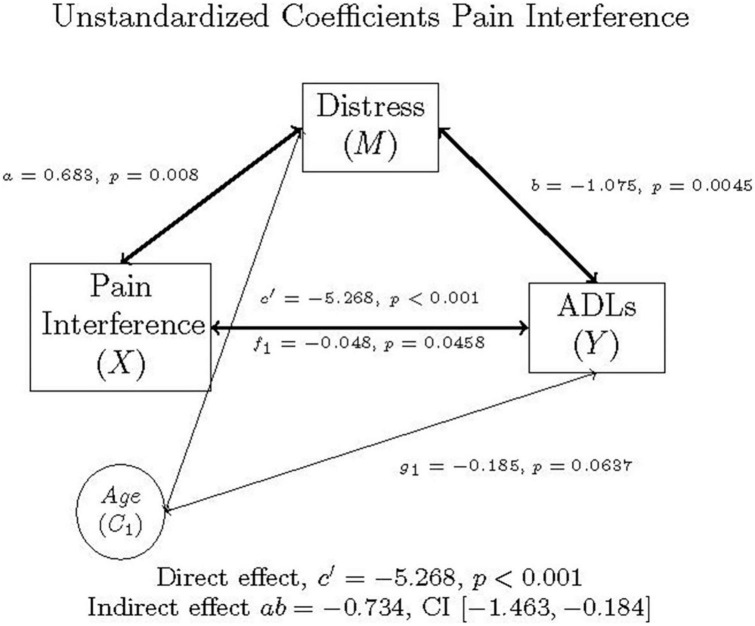
Mediation model of pain interference, distress, ADLs, and age in individuals with FM, as well as the direct effect, indirect effect, and confidence interval using unstandardized (b) coefficients.

The second mediation model mirrored the first, replacing pain intensity with pain interference (*x*). In Figure 2, the (*x*) is pain interference (the independent variable), (*y*) is ADLs (the dependent variable). The *c* path is the correlation between pain interference and ADLs, controlling for age in individuals with FM.

The PROCESS output is summarized in Tables 7, 8, and graphically represented in Figure 2. As with Model 1, mediation occurred in Model 2, as well.

**TABLE 7:** Model coefficients for the pain interference, distress, ADLs simple mediation model with covariate of age.

Antecedent		Consequent								
		*M* (Distress)					*Y* (ADLs)			
		β	*b*	*SE*	*p*		β	*b*	*SE*	*p*
X (pain interference)	*a*	0.275	0.683	0.2139	0.008	*C’*	–0.4598	–5.268	0.906	<0.001
M (distress)		−	−	-	−	*b*	–0.2326	–1.075	0.371	0.0045
C_1_ (age)	*f*_1_	–0.174	–0.048	0.0240	0.0458	*g*_1_	–0.1445	–0.185	0.099	0.0637
Constant	*i*_*M*_	−	4.2379	1.6997	0.014	*i*_*Y*_	−	68.233	7.086	<0.001
		*R*^2^ = 0.1161					*R*^2^ = 0.3218			
		*F*(2, 120) = 7.8783, *p* = 0.0006					*F*(3, 119) = 18.8225, *p* < 0.001			

**TABLE 8:** Mediation effects of distress on the relationship between pain interference and ADLs controlling for age, *N* = 123.

			95% CI	
Effect	β	*b*	Lower	Upper
Total (*c* path)	–0.524	–6.002	–7.7763	–4.2281
Direct (*c*’ path)	–0.460	–5.268	–7.0617	–3.4745
Indirect (mediation, *ab* path)	–0.064	–0.7342	–1.4627	–0.1841

Multiplying *a* and *b* yields the indirect effect, *ab* = 0.683 (-1.075) = –0.7342. A 95% bootstrap confidence interval was used and did not cross zero. The direct effect was *c’* = –5.268. The total effect was derived by adding the direct and indirect effects: *c* = *c’* + *ab* = –5.268 + –0.7342 = –6.002. This was all while controlling for age. The confidence intervals did not cross zero, and everything was significant, suggesting that the model is considered a good fit for the data. As before, a Sobel Test was also conducted, which indicated that mediation did not occur (Test statistic = 1.890, *p* = 0.0588), though Hayes (58) recommends the 95% bootstrapped confidence intervals instead be used to determine model fit and mediation.

## Discussion

The results indicate that depression and anxiety (i.e., the symptom cluster of distress) influence the relationship between pain intensity, pain interference, and ADLs while controlling for age in individuals with FM. This adds to the existing literature by looking at a chronic pain specific sample in Orange County, California, by including pain interference as well as pain intensity, and by calculating a distress variable in the analyses using both anxiety and depression.

The overall mediation models correspond with previous findings, in that the two aspects of pain and distress are negatively and significantly related to ADLs, and that age also plays a part in these relationships. Mediation occurred in both Model 1 and Model 2. This means that in Model 1, the symptom cluster of distress (anxiety and depression) mediated the relationship between pain intensity and ADLs, while controlling for age in a sample of FM individuals. In Model 2, the symptom cluster of distress (anxiety and depression) mediated the relationship between pain interference and ADLs, while controlling for age in a sample of FM individuals. In other words, depression and anxiety impact the functionality of those with FM when those afflicted are also experiencing pain.

These results coincide with previous findings. Steiner et al. (47) found that depression was a partial mediator of the relationship between physical functioning (ADLs) and pain intensity for their sample at all four time points. In this study, higher levels of depression were related to lower levels of ADLs and higher levels of pain, suggesting that treating depression could be a viable option in the pursuit of improving pain symptoms and ADL abilities for those with FM.

We retested the mediation relationship that Steiner et al. (47) found while using measures specifically validated for use in older adults with FM (i.e., the BDI-II for depression and the PAI for ADLs) to see whether the relationship still exists. We also included the important variable of anxiety and controlled for age. Our findings coincided with those of Steiner et al. (47), indicating that emotion or mood should be treated while attempting to improve physical functioning in older adults with FM.

Additionally, depression and anxiety, constructs that make up the symptom cluster of distress, are not normal parts of aging, although older adults do experience more life stressors that would cause distress like the loss of loved ones (59). Older adults are in fact less likely to be diagnosed with generalized anxiety disorder (GAD) than younger adults, and the rate of suicide in older adults is declining (18, 60). These factors may further explain why age was negatively related to the symptom cluster of distress in the sample.

What is interesting is that the relationships between age and some of the key variables were in the opposite direction than what was predicted. Specifically, those who were older were reporting lower levels of pain intensity and distress than their younger counterparts. Age had no significant relationships with ADLs or pain interference. This may be due to another variable that was not included in this study, such as time since diagnosis, level of education, or fatigue. These variables were not included in the *a priori* analyses due to sample size constraints, although *post hoc* analyses were performed.

### *Post hoc* analyses

#### Time since diagnosis

It can take on average 2.3 years to be diagnosed with FM after already experiencing years of symptoms, and even more time and testing to receive proper treatment (20, 61). During this process, those with FM who are not yet diagnosed are spending money, time, and energy while battling newfound and unwelcome symptoms. Delaying treatment and thus management of symptoms may make interventions less effective (6, 62, 63).

*Post hoc* analyses were run to determine whether time since diagnosis, and anxiety, depression, pain intensity, pain interference, and ADLs were related, and it was found that only pain intensity had a positive significant relationship with time since diagnosis. The relationship was small and positive while controlling for age. This indicates that those who were diagnosed some time ago have higher pain intensity than those who were diagnosed more recently, regardless of age.

#### Level of education

Level of education is also important to examine when studying FM (50, 52). *Post hoc* analyses were performed to determine whether level of education, and anxiety, depression, pain interference, pain intensity, and ADLs were related. Similar to time since diagnosis, level of education was only significantly related to pain intensity, but in a small, negative way while controlling for age. That is, those with higher levels of education such as college and beyond reported lower levels of pain intensity than those with lower levels of education.

#### Fatigue

Fatigue is another important variable that was not included in the original analyses due to lack of power (50, 52). *Post hoc* analyses were also performed to determine whether fatigue, and anxiety, depression, pain interference, pain intensity, and ADLs were related. It was found that fatigue was significantly positively related to anxiety, depression, pain interference, and pain intensity while controlling for age. Additionally, fatigue was significantly negatively related to ADLs while controlling for age. That is, regardless of age, the higher levels of fatigue an individual with FM experiences, the higher their anxiety, depression, pain interference, and pain intensity. Additionally, if their fatigue levels are lower, they are more able to perform ADLs. Results indicate that it might be beneficial to include the above variables when researching this topic in the future.

### Limitations

The limitations of this study include sample size and limited generalizability. The sample only included first time participants from 2008 to 2014, and only those who had FM. This means that the sample was *N* = 123, and unfortunately too small to include variables like time since diagnosis, level of education, and fatigue in the model. Also, the sample was primarily white, female, and most had an income higher than $50,000 per year. The participants’ demographic makeup makes it difficult to generalize to the greater population. At CSUF, we are fortunate to have access to the Osher Lifelong Learning Institute (OLLI) on campus, where older adult participants may be recruited for studies. That does mean that portions of this sample may have a higher education relative to same-aged peers, and they may be more physically, mentally, and socially active. This was not controlled for in this study due to power constraints, but should be controlled for in future studies. Additionally, anxiety, pain intensity, and pain interference were only assessed with one question, though the items have been validated using FM samples.

### Future directions

Future investigations should use a larger sample size and compare those with FM against those without FM, to see if the relationships with key variables and age would remain the same. Additionally, due to the small sample size, the results could not confirm the relationship between certain variables due to lack of power. Specifically, time since diagnosis, level of education, and fatigue were not used in these models when theoretically they may have added to our understanding of the relationship between pain intensity, pain interference, distress, and ADLs (50, 52). In the future we would like to utilize a smaller, less intensive study that uses these variables specifically in an online survey, allowing for a larger sample size. There would be fewer in-depth measures, but we would capture more responses on specific variables. We would also like to look at distress, education, comorbidities, and antidepressants while considering ADLs and age.

### Clinical implications

One recommendation that might come from this research is that depression and anxiety should be examined when it seems like pain is interfering with ADLs, especially in older adults with FM. The practical implications of these findings are that in clinical practice as well as in treatment centers for chronic pain, distress must be addressed for older adults with FM to maintain optimal functionality. Either prior to or in tandem with a prescription for the FDA approved medications for FM, doctors should also assess anxiety and depression, and then look into treating those with high levels of these affective disorders. If the goal of health professionals is to improve functionality in the FM population, distress must be addressed in addition to pain and the other symptoms of FM.

#### Treatment

On the topic of treating pain, in a chronic pain condition like FM, many treatment modalities are used. As seen in Table 9, these include therapeutic or psychological treatments, as well as medical interventions. However, not all treatments are effective for all types of pain. Sturgeon (64) suggests that psychological treatments for chronic pain differ in efficacy because they also vary in breadth, duration, and type. Some psychological therapies used to treat chronic pain conditions like FM include: operant-behavioral therapy, cognitive-behavioral therapy (CBT), mindfulness-based therapy and, most recently, acceptance and commitment therapy. Additionally, those with pain are not homogeneous, and we do need specific treatments for specific groups and for those who have different scores in different symptom clusters (11). Accordingly, psychological therapy is used to help treat chronic pain conditions and should be suggested to older adults with FM who are exhibiting symptoms of the cluster distress in addition to medical interventions.

**TABLE 9:** Management of FM/chronic pain.

Studies	Type of article	Focus	Outcomes
(78)	Systematic review/Meta-analysis in chronic widespread pain	Self-management RCTs including exercise and a psychological component	Some evidence for better physical function and less pain after treatment at 3 and 6 months.
(6)	Review of fibromyalgia and potential treatment options	What is and isn’t know about FM and treatment	Recommend psychosocial treatment with a combination of physical exercise and non-pharmacological therapies
(88)	Review of fibromyalgia and potential treatment options	What is and isn’t know about FM and treatment	Recommend multidisciplinary management of symptoms as well as medication.
(89)	Review of fibromyalgia and potential treatment options	Pharmacological treatments Psychosocial treatments: Acupuncture, electric stimulation, vibroacoustic and rhythmic sensory stimulation, hyperbaric, laser therapy, phototherapy, exercise, and massage, probiotics therapy, plant extracts	Pharmacologic and alternative therapies best.
(70)	Meta-analysis of fibromyalgia and trans-magnetic stimulation (TMS)	Evaluating TMS as a diagnostic criterion for FM as well as for a treatment option.	Evidence for unbalanced inhibitory-excitatory regulation in motor cortex. Inhibitory dysfunction may be moderated by exercise, certain medications, and electrical brain-stimulation, resulting in less pain.

As Okifuji and Hare (65) point out, the treatment of FM and its comorbid conditions usually includes a combination of medication, therapy and exercise. Common medications used to treat FM are pregabalin, duloxetine, and milnacipran (65, 66). Pregabalin was approved by the Food and Drug Administration (FDA) in 2007 and is a γ-aminobutyric acid (GABA) analog and antiepileptic agent. Duloxetine was approved by the FDA in 2008 and milnacipran was approved by the FDA in 2009. Both duloxetine and milnacipran are serotonin-norepinephrine reuptake inhibitors (SNRIs).

In addition to the treatments described above, there are numerous over the counter medications, off brand medications, and alternative therapies that have shown promise in alleviating symptoms of fibromyalgia and its comorbidities. This brief list is not comprehensive, but treatments of note are the Transcutaneous Electrical Nerve Stimulation unit, antihistamines, and low dose naltrexone (67–70). While there are theories as to the nature of FM, the underlying cause is still largely unknown, so treatment of the symptoms and comorbid conditions is key (71, 72).

Furthermore, Margolis et al. (73) studied resource utilization in FM (*N* = 64,038) and found that 18.7% used SNRIs like duloxetine and milnacipran, 27.4% used antiepileptic drugs like pregabalin, and the most commonly used drugs were opioids at 44.3%. Dussias et al. (74) reported that the most frequently prescribed pharmacological treatment for FM was antidepressants (46%).

In a study of 240,144 patients, only 31% (*n* = 74,738) were on a prescription medication in line with the ACR guidelines for FM. They also found that discontinuation, switching, and addition of new pain medications were common, which may indicate dissatisfaction with the initial treatment they were receiving. While on medication, FM patients still report pain, stiffness, fatigue, sleep problems and a myriad of other symptoms that medications do not completely treat, and so many patients look toward therapy and exercise to fill the gaps (6, 75, 76). Illustrating this lack of efficacy (limited pain relief and a number of side effects), in Germany there has been a big push away from pharmaceuticals and toward non-pharmacologic treatments of FM in recent years (6).

Non-pharmacological therapies used to treat FM and similar chronic pain conditions include CBT, emotional awareness and expression therapy, acceptance and commitment therapy, mind-body therapies (MBT), lifestyle, and medical education (77–80). It is recommended that any type of therapy be accompanied by exercise for FM patients, since exercise seems to help most FM symptoms and has the fewest side effects (81, 82). The most recommended exercise for FM treatment is aerobic exercise, while the least recommended treatment is resistance, but nearly any type of exercise has been shown to help treat FM symptoms (83, 84). Activities like yoga, tai chi, and strength training have also been shown to improve FM symptoms, although it can be particularly difficult for people with FM to adhere to an exercise regimen because initially, exercise can make the pain worse (85, 86). Recent studies have explored trans-magnetic stimulation as a possible treatment option for FM as well (70). As with any chronic pain condition, while the comorbid conditions and symptoms of FM can be managed to some extent, even with pharmaceutical and non-pharmacologic treatment, the detrimental effects of the syndrome may persist (87). This is why screening for and treating distress in older adults with FM is essential. Treating mood may help treat the other symptoms.

## Conclusion

This research was important because depression and anxiety measures are not yet being used when examining individuals with FM who are older, and they should be a part of intake at appointments in order to get a more expansive picture of the issue at hand, and to help those with FM develop a more comprehensive treatment plan more quickly.

Drugs and other pharmaceuticals often are ineffective and under-prescribed for the majority of individuals with FM. Of those who are finally receiving treatment and taking medications, after potentially years of attempting to receive a diagnosis, the adherence rate is low. Those who have a longer time since diagnosis seem to be managing their pain less effectively, which could affect their ADLs and distress scores. Other treatment modalities and further research are therefore imperative, and may be even more important than ever in the wake of the pandemic. There is a rise in myalgic conditions like FM, specifically Long COVID, and those affected by this are having a strikingly similar experience to those with FM when seeking treatment and a diagnosis.

## Data availability statement

The data supporting the findings of this study are available from the corresponding author LZ-W, lzettel-watson@fullerton.edu on request.

## Ethics statement

The studies involving human participants were reviewed and approved by the California State University, Fullerton, Institutional Review Board. The patients/participants provided their written informed consent to participate in this study.

## Author contributions

All authors listed have made a substantial, direct, and intellectual contribution to the work, and approved it for publication.
